# Predictive modeling for acute kidney injury after percutaneous coronary intervention in patients with acute coronary syndrome: a machine learning approach

**DOI:** 10.1186/s40001-024-01675-0

**Published:** 2024-01-24

**Authors:** Amir Hossein Behnoush, M. Moein Shariatnia, Amirmohammad Khalaji, Mahsa Asadi, Alireza Yaghoobi, Malihe Rezaee, Hamidreza Soleimani, Ali Sheikhy, Afsaneh Aein, Somayeh Yadangi, Yaser Jenab, Farzad Masoudkabir, Mehdi Mehrani, Mina Iskander, Kaveh Hosseini

**Affiliations:** 1 Cardiac Primary Prevention Research Center, Cardiovascular Diseases Research Institute, Tehran University of Medical Sciences, Tehran, Iran; 2 Tehran Heart Center, Cardiovascular Diseases Research Institute, Tehran University of Medical Sciences, Tehran, Iran; 3 School of Medicine, Tehran University of Medical Sciences, Tehran, Iran; 4 Non-Communicable Diseases Research Center, Endocrinology and Metabolism Population Sciences Institute, Tehran University of Medical Sciences, Tehran, Iran; 5 Department of Pharmacology, School of Medicine, Shahid Beheshti University of Medical Sciences, Tehran, Iran; 6 Department of Cardiovascular Medicine, Medical College of Wisconsin, Milwaukee, WI USA

**Keywords:** Acute coronary syndrome, Percutaneous coronary intervention, Acute kidney injury, Machine learning, Prediction

## Abstract

**Background:**

Acute kidney injury (AKI) is one of the preventable complications of percutaneous coronary intervention (PCI). This study aimed to develop machine learning (ML) models to predict AKI after PCI in patients with acute coronary syndrome (ACS).

**Methods:**

This study was conducted at Tehran Heart Center from 2015 to 2020. Several variables were used to design five ML models: Naïve Bayes (NB), Logistic Regression (LR), CatBoost (CB), Multi-layer Perception (MLP), and Random Forest (RF). Feature importance was evaluated with the RF model, CB model, and LR coefficients while SHAP beeswarm plots based on the CB model were also used for deriving the importance of variables in the population using pre-procedural variables and all variables. Sensitivity, specificity, and the area under the receiver operating characteristics curve (ROC-AUC) were used as the evaluation measures.

**Results:**

A total of 4592 patients were included, and 646 (14.1%) experienced AKI. The train data consisted of 3672 and the test data included 920 cases. The patient population had a mean age of 65.6 ± 11.2 years and 73.1% male predominance. Notably, left ventricular ejection fraction (LVEF) and fasting plasma glucose (FPG) had the highest feature importance when training the RF model on only pre-procedural features. SHAP plots for all features demonstrated LVEF and age as the top features. With pre-procedural variables only, CB had the highest AUC for the prediction of AKI (AUC 0.755, 95% CI 0.713 to 0.797), while RF had the highest sensitivity (75.9%) and MLP had the highest specificity (64.35%). However, when considering pre-procedural, procedural, and post-procedural features, RF outperformed other models (AUC: 0.775). In this analysis, CB achieved the highest sensitivity (82.95%) and NB had the highest specificity (82.93%).

**Conclusion:**

Our analyses showed that ML models can predict AKI with acceptable performance. This has potential clinical utility for assessing the individualized risk of AKI in ACS patients undergoing PCI. Additionally, the identified features in the models may aid in mitigating these risk factors.

**Graphical Abstract:**

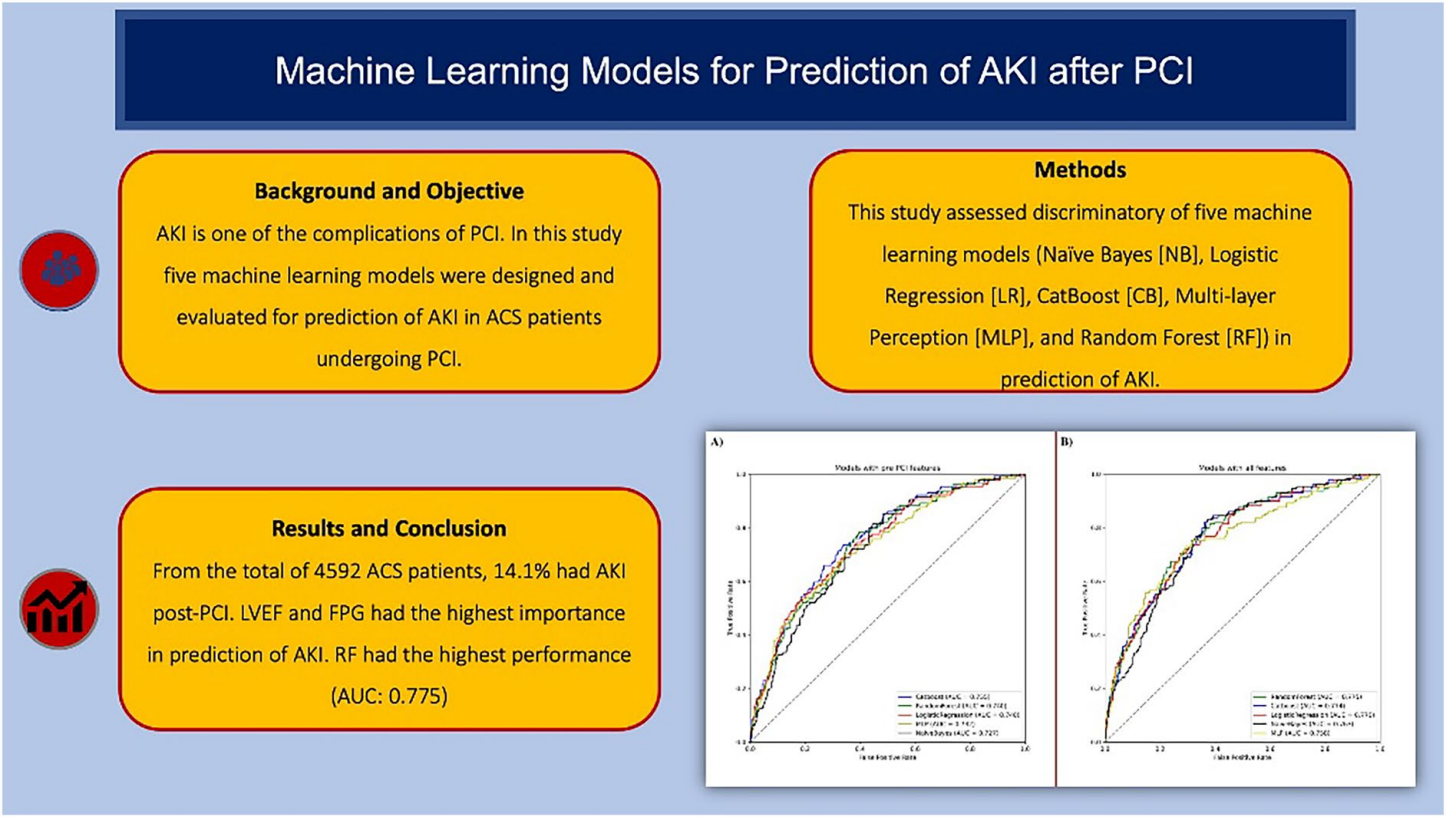

## Introduction

Coronary artery disease, particularly acute coronary syndrome (ACS), is responsible for approximately one-third of all deaths in adults over 35. Nowadays percutaneous coronary intervention (PCI) is the most widely used treatment for ACS. Acute kidney injury (AKI) is a serious non-cardiovascular complication in patients with ACS, and nearly 12.8% of the patients develop AKI as a major post-PCI complication with a 20.2% attributed mortality rate during or after hospitalization [1, 2]. A growing body of evidence indicates that AKI is significantly associated with an increased risk of long-term morbidities such as repeated coronary revascularization, myocardial infarction, and stroke [3, 4].

To prevent contrast induced-AKI (CI-AKI), physicians can implement preventive measures such as regulating contrast volume and osmolarity, pre-procedural statin intake, and pre- and post-procedural hydration [1, 5]. Identifying PCI-related patient risks allows physicians to tailor strategies based on each individual’s risk profile, leading to fewer complications and improved clinical outcomes after a PCI procedure [1, 6]. Prediction models, such as the NCDR-AKI risk model, have been developed to assess the risk of CI-AKI prior to performing PCI with a c-statistics of 0.71 [7]. Traditional statistical models may not include all possible interactions when there are numerous candidate variables, resulting in a decrease in the model’s accuracy when these interactions are ignored [1, 8]. Machine Learning (ML)-based models do not depend on assumptions about the variables involved or their relationship with the outcome. Instead, they capture complex relationships in a data-driven manner, including nonlinearity and interactions that may be difficult to identify otherwise. These models have been used for the prediction of outcomes in cardiovascular medicine [9–11].

This study aims to evaluate novel ML-based models to more accurately predict the risk of PCI-induced AKI in ACS patients and subsequently reduce the risk of long-term complications. The efficacy of ML-based models will be compared with traditional stepwise selection models, and the study will investigate whether machine learning-based models can sufficiently reduce the variables needed for disease prognosis prediction.

## Methods

### Study design

We retrospectively reviewed all patients with ACS [ST-elevation myocardial infarction (STEMI), non-STEMI, and unstable angina (UA)] who underwent PCI at Tehran Heart Center between 2015 and 2020. The ethics committee of Tehran Heart Center approved this study (IR.TUMS.MEDICINE.REC.1402.178). The informed consent was waived due to the retrospective design of this study.

### Variable’s definition and outcome

Pre-procedural variables used were: gender, age, left ventricular ejection fraction (LVEF), atrial fibrillation (AF), fasting plasma glucose (FPG), triglycerides (TG), total cholesterol (TC), low-density lipoprotein cholesterol (LDL-C), high-density lipoprotein cholesterol (HDL-C), drug history (lipid-lowering, anti-diabetes, anti-hypertension, anti-arrhythmia, and anti-thrombotic), hematocrit, body mass index (BMI), estimated glomerular filtration rate (eGFR), creatinine (Cr), type of diabetes management, past medical histories (cardiac, renal, previous PCI, previous CABG), and CAD risk factors.

Procedural variables were: non-ST elevation myocardial infarction (NSTEMI) in coronary angiography (CAG), acute MI in CAG, treated vessel, procedure result, stenosis, stent diameter, stent length, stent inflation pressure, post-procedural complications (arrhythmia, cardiopulmonary resuscitation (CPR), aborted cardiac arrest, and procedure-induced shock).

AKI, the primary outcome in this study, was defined based on the acute kidney injury necrosis (AKIN) as an absolute increase of ≥ 0.3 mg/dL or a relative increase of ≥ 50% in serum creatinine after the procedure [12].

### Data cleaning

At first, patients with missing data for follow-up were removed and missing data for other features were handled through imputation with median values for numerical features and mode for categorical ones. Notably, features with more than 40% missing data were removed from the models. Then, the patients with end-stage renal disease (ESRD) (eGFR < 15 mL/min) were excluded. Moreover, we excluded individuals with implausible creatinine values (Cr < 0.3 mg/dL or Cr > 4.0 mg/dL). Label encoder (from the scikit-learn library) was used to change categorical variables into numerical variables.

### Train/test split and feature selection

We randomly assigned each patient to the train (80%) or test (20%) dataset using stratified splitting. Five-fold cross-validation was used in this study for feature selection and hyperparameter tuning. To find the most important variables among the vast number of procedural and post-procedural features, and to reduce the complexity of our models, we first trained an RF model on these features from our training dataset as our feature selector. We selected the top 15 features based on the feature importance given by this model. This cutoff was defined as we wanted to use features, sum of which contributed to 80% of the total feature importance. The selected features are used as our procedural features to train the main models in this study. Moreover, SHapley Additive ExPlanations (SHAP), as a game-based feature analysis technique [13], based on the CatBoost model were used to generate beeswarm plots for feature importance.

We feature-engineered a few variables to provide the models with more context and useable features. As there were multiple creatinine measurements before the PCI procedure, we added creatinine standard deviation, creatinine mean, and creatinine change (defined as the difference between the last creatinine before PCI and the first creatinine levels), in addition to the last creatinine measurement before PCI for each patient.

Moreover, as there were multiple binary features for each patient encoding various past medical conditions, risk factors, and drug history, we defined the new features to reduce the complexity of our models. This was based on the fact that in most cases there was not a high number of cases positive for each feature and by merging them, the models could take advantage of variables with a higher proportion of positive cases. The following features were used: (i) cardiac risk factors which are defined as the number of all cardiac risk factors each patient had (family history, hyperlipidemia, diabetes, and hypertension); (ii) cardiac past medical history (PMH), which is defined as the number of cardiac conditions (STEMI, NSTEMI, CHF, PMH valvular heart disease, peripheral vascular disease, and CPR), renal PMH (renal failure, and dialysis); (iii) other PMH (SA, UA, chronic lung disease); (iv) anti-arrhythmic, which is defined as the number of all anti-arrhythmic medications the patient has been using (Digoxin and Amiodarone); (v) anti-thrombotic and platelet (Aspirin, Clopidogrel, and Warfarin); (vi) anti-hypertension (angiotensin receptor blockers, beta-blockers, calcium channel blockers, and diuretics); and (vii) diabetes medications (metformin, glibenclamide, acarbose, and insulin).

### Model development

We used five models to predict AKI in patients who underwent PCI: Naïve Bayes (NB), logistic regression (LR), CatBoost (CB), multilayer perception (MLP), and Random Forest (RF). Each model was trained and evaluated using five-fold cross-validation. Test data remained unseen during model development.

### Model evaluation

Models were evaluated using three metrics: area under the receiver operating characteristics curve (AUC), sensitivity, and specificity. The AUC is independent of the threshold and measures the discriminative ability of models by plotting the true positive rate against the false positive rate. Different AUC scores could be categorized as follows: (1) outstanding (AUC ≥ 0.9), (2) excellent (0.8 ≤ AUC < 0.9), (3) acceptable or fair (0.7 ≤ AUC < 0.8), (4) poor (0.6 ≤ AUC < 0.7), and no discrimination (AUC < 0.6). Finally, to make the RF model explainable and assess the effect of each variable on overall predictive ability, we used an explainable AI method on positive and negative individual cases separately, to gain more insight into the effect of each feature on the final probability predicted by the RF model [14].

### Statistical analysis

Mean ± standard deviation or proportion (percentage) was used for reporting baseline characteristics of patients who developed and did not develop AKI in each of the test and train cohorts. Regarding AUCs, we calculated the 95% confidence interval (CI) of AUCs using a 1000-time bootstrap in the test cohort. We developed the models and performed all the analyses using Python (version 3.8). LR, RF, MLP, and NB models were trained using the scikit-learn (1.0.2) library [15] and CB using the CatBoost library (version 1.2) Python library.

## Results

### Patient characteristics

A total of 4592 patients were identified among which 646 (14.1%) had developed AKI after undergoing PCI. The train data consisted of 3672 (80%) patients (517 had AKI), and the test data included 920 cases (20%), among which 129 had AKI. Mean age of the total population was 65.6 ± 11.2 years (69.2 ± 11.6 in the AKI group and 65 ± 11.2 in the non-AKI group) and males contributed to 73.1% of the overall cohort. Moreover, with a mean BMI of 28.1 ± 4.4 kg/m^2^, 57.7% had hypertension, 44.9% were diabetic, and 39.1% were smokers. Recorded data showed that 82.3% of patients with AKI and 63.1% of those without had acute MI (STEMI or NSTEMI). Details of all baseline characteristics and angiography data for the test and train cohort are shown in Table 1.

**Table 1:** Baseline characteristics of patients who developed and not developed AKI in train and test cohorts

	Train cohort		Test cohort	
	AKI (*n* = 517)	No AKI (*n* = 3155)	AKI (*n* = 129)	No AKI (*n* = 791)
Age (years)	69.5 ± 11.5	65 ± 11.2	69.5 ± 12.1	64.9 ± 11.1
Sex (male)	352 (68.1%)	2352 (74.5%%)	89 (69%)	566 (71.6%)
BMI (kg/m^2^)	28.1 ± 4.6	28.1 ± 4.4	28.8 ± 4.6	28.1 ± 4.4
Waist circumference (cm)	100.7 ± 11.1	100.2 ± 10.5	101.4 ± 9.8	99.8 ± 10.7
Hypertension	334 (64.6%)	1782 (56.5%)	87 (67.4%)	448 (56.6%)
Diabetes	311 (60.1%)	1321 (41.9%)	74 (57.4%)	358 (45.2%)
Cigarette smoking	172 (33.3%)	1283 (40.7%)	45 (34.9%)	296 (37.4%)
Heart failure	51 (9.9%)	138 (4.4%)	19 (14.7%)	32 (4%)
Atrial fibrillation	15 (2.9%)	37 (1.2%)	4 (3.1%)	8 (1%)
Valvular heart disease	26 (5%)	84 (2.7%)	3 (2.3%)	19 (2.4%)
Peripheral vascular disease	1 (0.2%)	15 (0.5%)	1 (0.8%)	3 (0.4%)
Chronic lung disease	15 (2.9%)	67 (2.1%)	1 (0.8%)	19 (2.4%)
Previous PCI	82 (15.9%)	509 (16.1%)	20 (15.5%)	130 (16.4%)
Previous CABG	74 (14.3%)	402 (12.7%)	19 (14.7%)	115 (14.5%)
History of CVA	37 (7.2%)	103 (3.3%)	11 (8.5%)	33 (4.2%)
Opium	66 (12.7%)	496 (15.7%)	18 (13.9%)	102 (12.9%)
History of STEMI	36 (7%)	188 (5.9%)	5 (3.9%)	34 (4.3%)
History of NSTEMI	46 (8.9%)	428 (13.6%)	23 (17.8%)	113 (14.3%)
History of UA	60 (11.6%)	831 (26.3%)	10 (7.7%)	194 (24.5%)
History of SA	4 (0.8%)	49 (1.5%)	2 (1.5%)	18 (2.3%)
LVEF (%)	38.9 ± 10.2	43.8 ± 8.8	38 ± 10.1	44.3 ± 8.7
Total cholesterol (mg/dL)	151.9 ± 43.2	155 ± 41.8	152.8 ± 44.8	154.4 ± 42.1
Triglyceride (mg/dL)	134.7 ± 84.1	151.5 ± 98.1	134.2 ± 81	150.8 ± 97.8
LCL-C (mg/dL)	94.4 ± 35.2	96.6 ± 34.9	96.3 ± 37.1	96 ± 35.5
HDL-C (mg/dL)	39.2 ± 10.1	38.4 ± 9.6	39.9 ± 10.7	38.5 ± 9.6
FPG (mg/dL)	155.1 ± 78.6	130.1 ± 56.8	153.1 ± 76.3	134.9 ± 59.7
Last creatinine before PCI (mg/dL)	1.22 ± 0.56	1.09 ± 0.34	1.21 ± 0.48	1.09 ± 0.35
Hemoglobin (g/dL)	14 ± 2.2	14.6 ± 1.9	13.9 ± 2.3	14.4 ± 1.9
PCI and angiographic findings				
Acute MI (STEMI and NSTEMI)	428 (82.8%)	1980 (62.7%)	104 (80.6%)	508 (64.2%)
NSTE-ACS	170 (32.9%)	1404 (44.5%)	50 (38.8%)	446 (56.4%)
CPR	8 (1.5%)	46 (1.5%)	2 (1.5%)	9 (1.1%)
Lesion length (mm)	27.3 ± 14	27.3 ± 14.2	28.9 ± 13.8	26.9 ± 14
Pre-procedure stenosis (%)	92.8 ± 8.9	92.9 ± 8.3	95.3 ± 10.6	92.7 ± 8.8
Pre-procedure TIMI flow				
0	233 (45.1%)	1031 (32.7%)	69 (53.5%)	245 (31%)
1	25 (4.8%)	139 (4.4%)	9 (7%)	39 (4.9%)
2	84 (16.2%)	474 (15%)	19 (14.7%)	117 (14.8%)
3	174 (33.6%)	1511 (47.9%)	32 (24.8%)	390 (49.3%)
Vessel severity				
Single vessel	111 (21.5%)	798 (25.3%)	22 (17.1%)	189 (23.9%)
Two vessel	151 (29.2%)	1165 (36.9%)	42 (32.6%)	285 (36%)
Three vessel	251 (48.6%)	1185 (37.6%)	65 (50.4%)	315 (39.8%)
ACC/AHA category				
A	0 (0%)	5 (0.2%)	0 (0%)	3 (0.4%)
B1	47 (9.1%)	449 (14.2%)	6 (4.6%)	129 (16.3%)
B2	74 (14.3%)	430 (13.6%)	21 (16.3%)	100 (12.6%)
C	396 (76.6%)	2270 (71.9%)	102 (79.1%)	559 (70.7%)
PCI location				
Proximal	185 (35.8%)	1203 (38.1%)	45 (34.9%)	289 (36.5%)
Non-proximal	247 (47.8%)	1567 (49.7%)	63 (48.8%)	405 (51.2%)
Ostial	85 (16.4%)	385 (12.2%)	21 (16.3%)	97 (12.3%)

Data are represented as mean ± standard deviation, or number (%)

*BMI* body mass index, *PCI* percutaneous coronary intervention, *CABG* coronary artery bypass grafting, *CVA* cerebrovascular accident, *STEMI* ST-elevation myocardial infarction, *UA* unstable angina, *SA* stable angina, *LVEF* left ventricular ejection fraction, *LCL-C* low-density lipoprotein cholesterol, *HCL-C* high-density lipoprotein cholesterol, *FPG* fasting plasma glucose, *NSTE-ACS* non-ST elevation acute coronary syndrome, *CPR* cardiopulmonary resuscitation, *TIMI* thrombolysis in myocardial infarction, *ACC* American College of Cardiology, *AHA* American Heart Association

### Feature importance

**Fig. 1:**
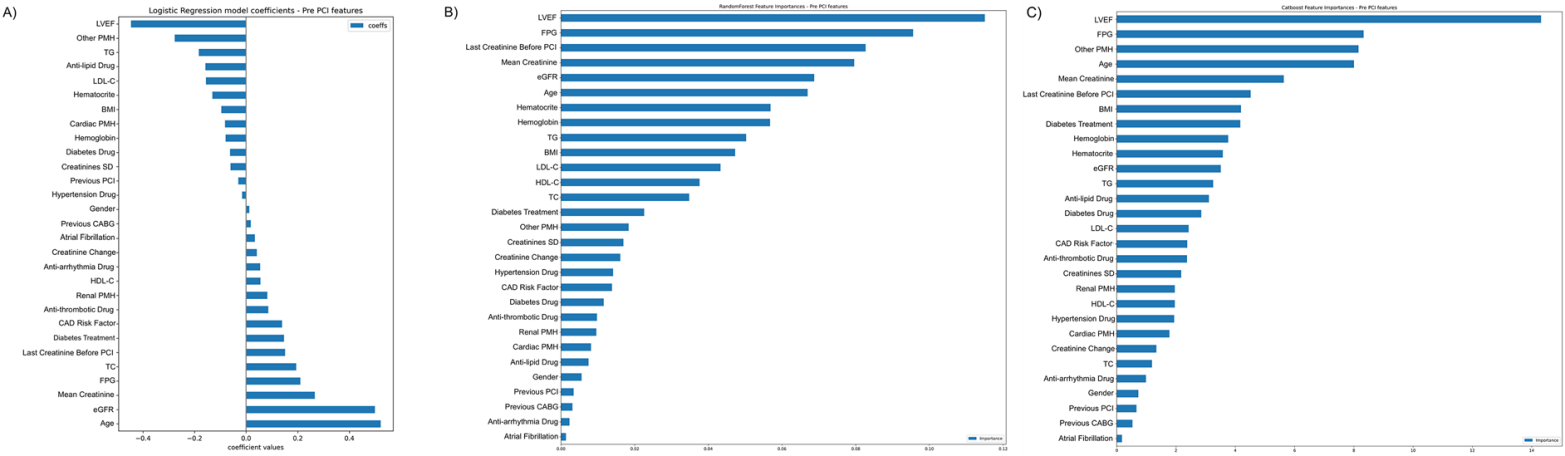
Feature importance for models using pre-procedural features only. **A** Logistic Regression Coefficients; **B** Random Forest Feature Importance; **C** CatBoost Feature Importance. *LVEF* left ventricular ejection fraction, *FPG* fasting plasma glucose, *CPR* cardiopulmonary resuscitation, *PCI* percutaneous coronary intervention, *BMI* body mass index, *TC* total cholesterol, *LDL-C* low-density lipoprotein cholesterol, *PMH* past medical history, *UA* unstable angina, *MI* myocardial infarction, *TG* triglyceride, *HDL-C* low-density lipoprotein cholesterol, *CAD* coronary artery disease, *CABG* coronary artery bypass grafting

**Fig. 2:**
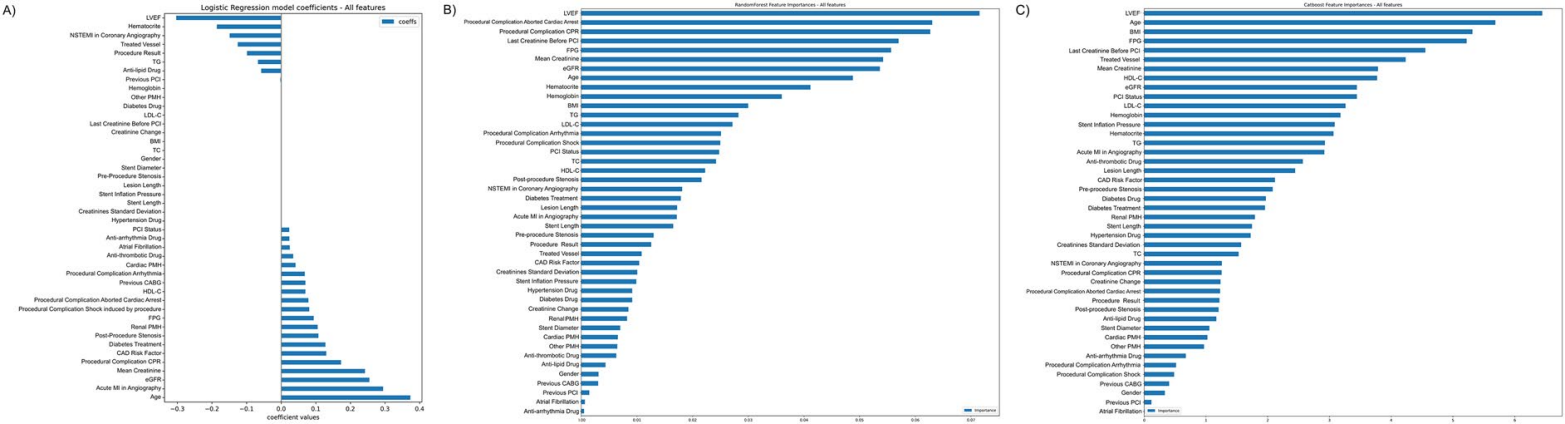
Feature importance for models using pre-procedural, procedural, and post-procedural features. **A** Logistic Regression Coefficients; **B** Random Forest Feature Importance; **C** CatBoost Feature Importance. *LVEF* left ventricular ejection fraction, *FPG* fasting plasma glucose, *CPR* cardiopulmonary resuscitation, *PCI* percutaneous coronary intervention, *BMI* body mass index, *TC* total cholesterol, *LDL-C* low-density lipoprotein cholesterol, *PMH* past medical history, *UA* unstable angina, *MI* myocardial infarction, *TG* triglyceride, *HDL-C* low-density lipoprotein cholesterol, *CAD* coronary artery disease, *CABG* coronary artery bypass grafting

**Fig. 3:**
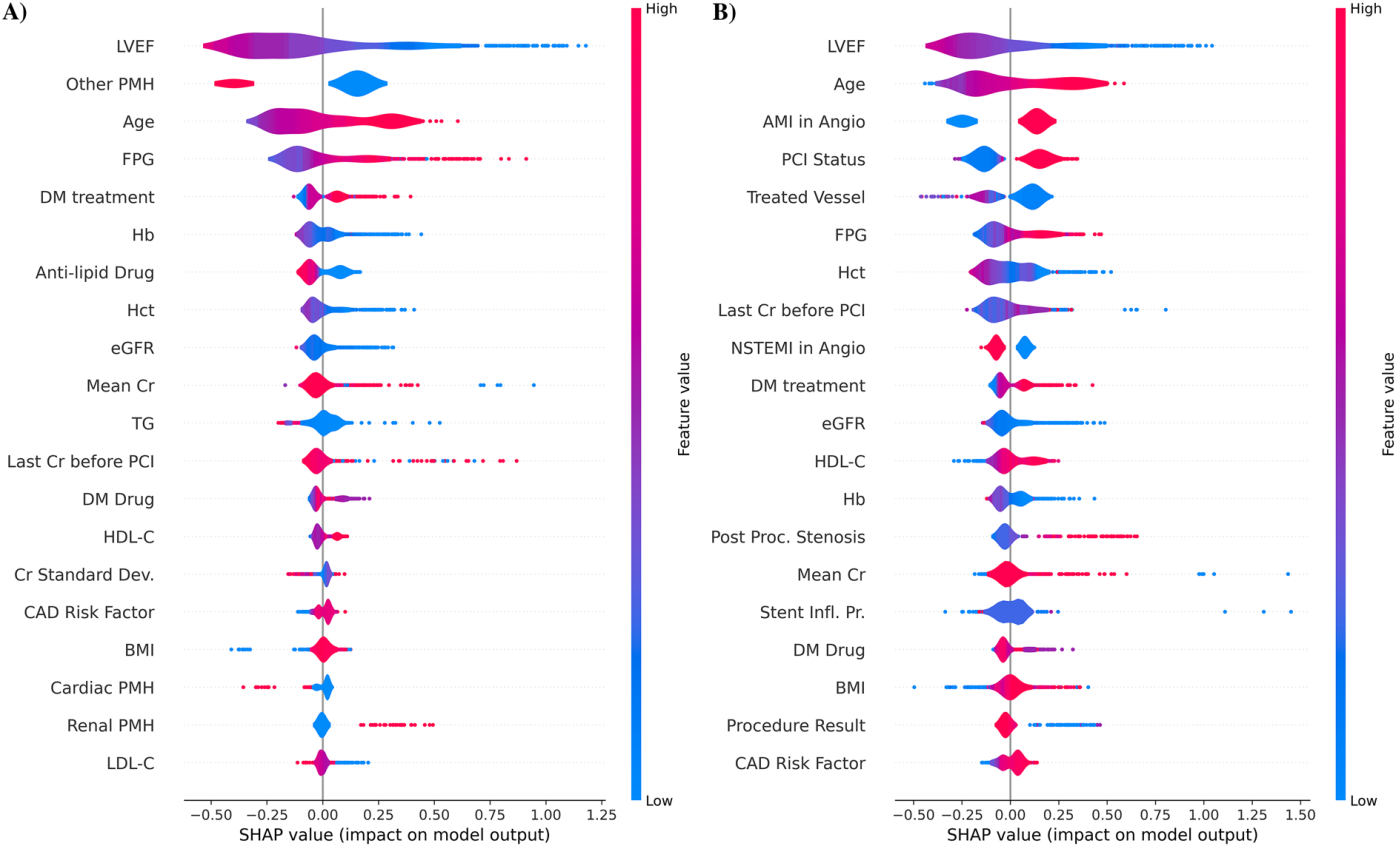
ROC-AUC Curves for the five ML models. **A** Using pre-procedure features only; **B** using pre-procedure and procedural features

Feature importance plots based on LR coefficients, RF model, and CB model for the prediction of AKI after PCI are shown using pre-procedural features in Fig. 1 and using all features in Fig. 2. The beeswarm plots for SHAP values based on the CB model are also illustrated in Fig. 3.

#### Pre-procedural features

Figure 1 shows feature ranks based on LR coefficients in addition to the RF and CB models using pre-procedure features only. As illustrated in Fig. 1A, LVEF had the highest negative correlation with AKI occurrence post-PCI, while age had the highest positive association with the outcome. RF model also ranked LVEF as the top feature, followed by FPG and the last creatinine before PCI (Fig. 1B). Finally, the CB model represented LVEF and FPG as the highest predictors of AKI post-PCI (Fig. 1C). Also, as shown in Fig. 3A as a SHAP beeswarm plot, LVEF ranked the highest classifier among the pre-procedural features.

#### Pre-procedural, procedural, and post-procedural features

LR model showed that LVEF was the main predictor of AKI with an inverse relationship between AKI and LVEF. However, age and acute MI in coronary angiography were the top positively correlated features, as depicted in Fig. 2A. Investigating the feature importance of the RF model, LVEF was the main predictor while aborted cardiac arrest in PCI, and CPR in PCI were the second and third top predictors (Fig. 2B). Last creatinine before PCI, FPG, mean creatinine, and eGFR were the next predictors. Figure 2C shows that LVEF, age, and BMI were the top three features, considering the CB model. Similarly, the SHAP beeswarm plot shown in Fig. 3B reports LVEF and age as the main discriminatory variables among all features.

### Model’s evaluation

**Fig. 4:**
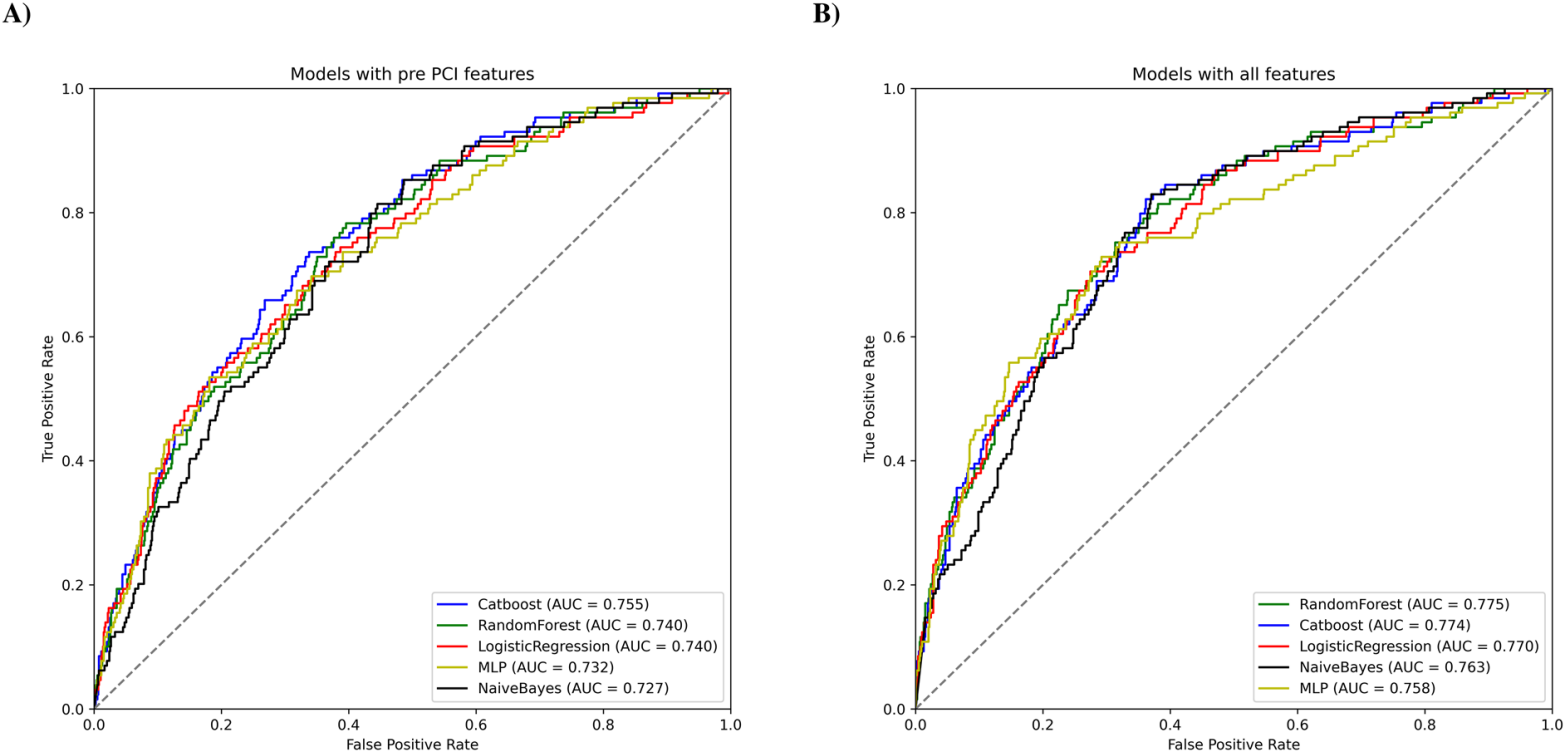
SHAP beeswarm plot for feature importance based on the CatBoost model. **A** Using pre-procedure features only; **B** using pre-procedure and procedural features

**Fig. 5:**
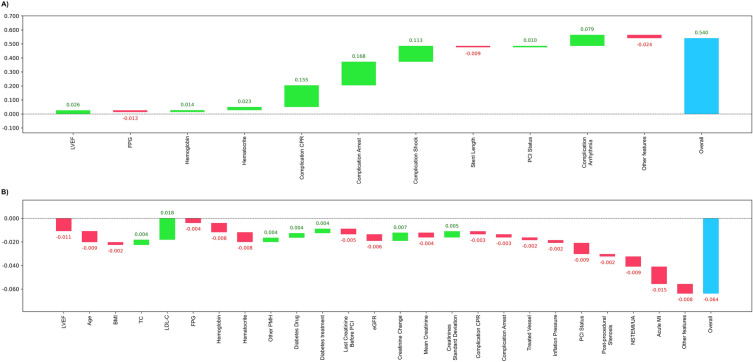
Effects of each variable on the RF model using pre-procedure, procedural, and post-procedural features for (**A**) one positive case (with AKI), **B** one negative case (without AKI). *LVEF* left ventricular ejection fraction, *FPG* fasting plasma glucose, *CPR* cardiopulmonary resuscitation, *PCI* percutaneous coronary intervention, *BMI* body mass index, *TC* total cholesterol, *LDL-C* low-density lipoprotein cholesterol, *PMH* past medical history, *UA* unstable angina, *MI* myocardial infarction

In this study five models were assessed for the prediction of AKI after PCI, the evaluation was once done using pre-procedural features only and once with pre-procedural, procedural, and post-procedural features, details are described in Table 2. In pre-procedural features-only analysis, the CB model outperformed the other models with an AUC of 0.755 (95% CI 0.713–0.797). RF and LR models had the same AUC of 0.74 (95% CI 0.694–0.783 and 0.689–0.785, respectively), followed by the MLP model with an AUC of 0.732 (95% CI 0.687–0.778) (Fig. 4A). In terms of sensitivity and specificity, RF and MLP recorded the highest, with 75.97% and 64.35% sensitivity and specificity, respectively. When procedural and post-procedural features were added to training features, the RF model performed the best in terms of AUC (0.775, 95% CI 0.730–0.818), slightly higher than the CB model (AUC 0.774, 95% CI 0.728–0.816). LR and NB models had AUCs of 0.770 (95% CI 0.725–0.811) and 0.763 (95% CI 0.715–0.804), respectively (Fig. 4B). A sensitivity of 82.95% in the CB model and a specificity of 82.93% in the NB model were the highest sensitivity and specificity among the models. With regard to making the final RF model explainable, as shown in Fig. 5, the effect of each feature in terms of raw probability is observed for one positive (5A) and one negative case (5B). The occurrence of the post-procedure arrest and CPR was effective in assigning a high probability for the positive cases by the RF model and the normal FPG of the patient was effective in reducing the overall probability. In negative cases, LDL-C and creatinine change had the highest negative impact while acute MI, NSTEMI/UA, and LVEF had the highest positive impact.

**Table 2:** Models’ evaluation for predicting AKI after PCI

	Pre-procedural features only			All features		
	Sensitivity	Specificity	AUC (95% CI)	Sensitivity	Specificity	AUC (95% CI)
Random Forest	75.97%	61.44%	0.740 (0.694–0.783)	81.4%	60.56%	0.775 (0.730–0.818)
Logistic Regression	75.19%	59.29%	0.740 (0.689–0.785)	76.74%	60.81%	0.770 (0.725–0.811)
Naïve Bayes	73.46%	57.9%	0.727 (0.682–0.768)	48.06%	82.93%	0.763 (0.715–0.804)
CatBoost	73.64%	64.1%	0.755 (0.713–0.797)	82.95%	62.58%	0.774 (0.728–0.816)
Multi-layer Perceptron	69.77%	64.35%	0.732 (0.687–0.778)	75.19%	65.36%	0.757 (0.708–0.804)

*AKI* acute kidney injury, *PCI* percutaneous coronary intervention, *AUC* area under the curve, *CI* confidence interval

## Discussion

In this registry-based study, we employed ML algorithms to predict the AKI incidence in ACS patients who underwent PCI. Our results indicated a novel step toward predicting AKI occurrence following PCI with the aid of ML, as all five ML models (RF, LR, CB, MLP, and NB) were able to enhance the prediction of AKI using both pre-procedural features only and all features combined data, with all models exhibiting an acceptable predictive capability (AUC value > 0.7), based on AUC interpretation [16]. While the CB model was the best when running the models with pre-procedural features only (AUC = 0.755), the RF model demonstrated the best performance using all features combined data (AUC = 0.775). The study also found that the RF model outperformed in terms of sensitivity (75.97%) and MLP had the highest specificity (64.35%) in the pre-procedural features analysis. Once procedural features were added, the CB model became the best model in terms of sensitivity (82.95%), while the NB model exhibited the highest specificity (82.93%).

As AKI is a preventable complication after PCI, a precise risk prediction system is needed which can improve clinical decision-making and management strategies such as sufficient hydration and contrast volume reduction in high-risk patients [7, 17]. The traditional models for the prediction of the risk of AKI after PCI have provided a notable enhancement in decision support and quality of care; however, they have several limitations and drawbacks, leading to underestimating the risk of AKI in a small proportion of patients, while overestimating it in others [18, 19]. For instance, relying on subjective assumptions for choosing the candidate variables, which are often transformed into categorical variables due to the convenience of calculating the risk scores [6]. Moreover, the single-center or single-country nature of these studies may lead to limited application in other clinical settings and highlight the need for localized models designed for other regions. Another limitation was the lack of control for renal protective medications and the lack of implementing procedural features in some [7]. Even though helpful, the conventional models are not sufficiently precise for Individual personalized evaluation and shared decision-making models in the modern medicine [1, 20]. Therefore, looking for more competent alternatives to traditional risk estimation models seems indispensable. Through this purpose, ML models are being developed rapidly regarding risk stratification of AKI for further safety considerations before, during, and after PCI. The computational discipline of ML-based methods allows the algorithm formulation into models capable of recognizing complex patterns or interactions when utilizing extensive data [8, 21]. By incorporating ML into the model development process, there is the potential to enhance the accuracy of the AKI risk stratification [6, 22]. In addition, the utilization of all available variables in ML modeling along with a permutation test for variable selection could contribute to promoting the performance of the ML modeling. Accordingly, ML models demonstrated superior performance compared to conventional models in terms of predictive performance and risk stratification of AKI for patients with PCI [6].

Also, a recent study conducted by Kuno et al. attempted to compare the conventional logistic regression prediction model with that of an ML model concerning the calculation of the risk of AKI after PCI. They used the light gradient boosting model (GBM) ML algorithm, along with Lasso and SHAP methods for variable selection. Their results indicated that the ML model provided comparable risk quantification accuracy using fewer variables than the logistic regression model [8]. Our study had several similarities and differences with this study. One of the key differences was that in contrast with Kuno et al. study, we designed our ML models both with and without operative variables which might be helpful in clinical settings when operative features can also be utilized. The predictive ability of our models was comparable with those of this study in terms of AUC. In this study, the LR model had an AUC of 0.755 in the test dataset, while in ours, LR achieved an AUC of 0.74 and 0.77 in the model with pre-procedure features and the model with all features, respectively. One of the other differences was that this study used a different feature selection method, first using Lasso through LR and then subsequently with SHAP based on GBM, however, we implemented the RF feature selector while demonstrating feature importance with LR coefficients, RF model, and CB model, in addition to SHAP beeswarm plots.

In line with our results, the cohort study of Sun et al. suggested that ML algorithms, particularly the RF model, can promote the accuracy of contrast-induced AKI risk stratification following acute myocardial infarction. They identified that the RF machine learning algorithm achieved the highest sensitivity of 71.9%, an accuracy of 73.5%, and an AUC of 0.82, which notably outperformed LR models [1]. In agreement with previous studies, our results revealed that the RF model has the best predictive ability. The RF model, constructed using an ensemble of multiple decision trees, can overcome the issue of overfitting in ML analysis, by aggregating decisions across a vast number of randomly generated trees [23, 24]. One key advantage of the RF model is its ability to reduce the number of variables, thereby simplifying the final model which could be explained more easily and reducing the risk of overfitting it on noisy features. Furthermore, highly correlated features do not cause multi-collinearity issues for RF models. Hence, due to the RF model being an inherent ensemble model (ensemble of lots of decision trees) and knowing that it is more robust to inter-correlated features, we can expect better performance in most cases where RF is used, however, it’s not a universal rule [25, 26].

However, in contrast to the finding of the present study, Niimi et al. demonstrated that the XGBoost ML model can significantly improve the discrimination value for predicting the risk of AKI following PCI (C-statistics of 0.84, *P* < 0.001) [27]. XGBoost was one of the ideal candidates to be trained in our study as well; however, since XGBoost needs a large dataset such as the one in Niimi et al. study, acceptable performance could not be obtained using preliminary analyses in our study. In contrast, we used the CatBoost model, similar to XGBoost but with a lower chance of overfitting on smaller datasets. In our study, this model had the highest performance when using pre-procedural features, showing its strength in the prediction of AKI.

In a more recent publication, the support vector machine (SVM) model showed the most outstanding AUC of 0.784 in terms of identifying the risk of contrast-induced nephropathy in elective PCI patients. The SVM model also outperformed logistic regression models [28]. Therefore, although currently available evidence suggests the potential efficacy of ML models for AKI risk prediction after PCI, selecting the optimal model has remained controversial. Additionally, it has been shown that in datasets with imbalanced data, such as ours, RF could outperform support vector machine (SVM) and XGBoost [29]. SVM is also a time-consuming algorithm that takes plenty of time to train the models. On the other hand, the K-nearest neighbor (KNN) is not able to show the differences in the predictive ability of features. Hence, these models are not routinely used by the newer studies and have been replaced by methods such as RF.

In this study, we report the feature importance of the RF model, the CB model, and the coefficients of the LR model, both with and without procedural findings. Although selecting the suitable features based on the RF model might lead to eventually the overall better performance of the RF model, it should be noted that RF feature selection has been shown to perform better than the two other classifiers (Boruta and Recursive Feature Elimination) [26]. In performing feature engineering, we combined some of the variables such as risk factors and creatinine levels to produce new variables. Mean and SD of creatinine levels measured at different time points before PCI might add some complexity to the models. We created this variable to use more of the data available for patients. Clinicians can benefit from these values if more than one creatinine level is available during the hospitalization course. However, this is not a limitation, since there were many patients with only one creatinine level for which the model was also optimized. With the use of electronic records, the mean and SD of all creatinine levels measured before PCI can be added to the model to enhance predictive ability.

Based on the LR model coefficients, LVEF and age had the highest negative and positive correlation with AKI occurrence post-PCI, in both pre-procedural and all features. The RF model also showed LVEF as the most important feature, followed by FPG and the last creatinine, mean creatinine, and eGFR using pre-procedural findings. Previously, several investigations showed that hypotension, intra-aortic balloon pump, congestive heart failure, chronic kidney disease, diabetes, age > 75 years, anemia, and volume of contrast (known as Mehran score) contributed to AKI incidence after PCI [18, 30], while several of them have been associated with major adverse events as well [31–33]. Also, the combination of age, serum creatinine, EF, and eGFR levels (calculated as ACEF-MDRD score) was found to be associated with the risk of AKI after PCI [34, 35]. Similarly, a study reported that contrast-induced AKI (CI-AKI) following PCI was observed more frequently in patients with diabetes, LVEF < 50%, older age, severe heart failure following acute MI, previous aspirin use, and higher ACEF-MDRD score [36]. Additionally, it has been suggested that reduced LVEF and heart failure could increase the risk of AKI in patients who underwent coronary artery bypass grafting [37]. So far, evidence has demonstrated that higher FPG levels can participate in impaired kidney function, leading to an increased risk of AKI while higher FPG levels are often associated with other risk factors for AKI, which can further increase the likelihood of developing AKI after PCI [38].

In a study, Lasso and SHAP methods in ML selected that ST-elevation MI, eGFR, age, preprocedural hemoglobin, non-ST-elevation MI/unstable angina, heart failure at admission, and cardiogenic shock as the pertinent predictor for AKI risk after PCI [8]. On the other hand, Ma et al. reported 11 important predictors of CI-nephropathy after PCI, including uric acid, peripheral vascular disease, cystatin C, creatine kinase-MB, hemoglobin, N-terminal pro-brain natriuretic peptide, age, diabetes, systemic immune-inflammatory index, total protein, and low-density lipoprotein, using SHAP method [28]. Also, age, serum creatinine level, and LVEF were among the top 20 ranked important variables concerning CI-AKI risk stratification after acute MI, using the Boruta ML algorithm [1].

Given the potential importance of AKI as an adverse event after PCI, models such as the ones investigated in this study can have clinical applications in the prediction of AKI post-PCI in patients with ACS, after further confirmation in larger studies. With implementing easy-to-use variables both pre-procedural and procedural, these ML-based models provided acceptable predictions. Our models showed similar prediction ability between models with and without procedural variables. It is of importance since intra-procedural features are dependent on the skill of the team performing PCI which makes it subjective and, hence, makes the inherent risk of patients less highlighted [18, 39]. Individualized risk stratification in predicting PCI can lead to better prevention of AKI after PCI. LVEF, age, and FPG were the main predictors of AKI which are easy to measure in patients with ACS admitted to PCI units. Clinicians could take advantage of these models for the prediction of AKI and therefore, provide better care for those at higher risk. These kinds of models could be used regionally or even internationally when assessed in different settings and on different populations.

Several limitations to our research need to be mentioned. Firstly, the single-center nature of our study could affect our findings. Furthermore, it is essential to consider the potential impact of not incorporating confounding variables. It is also important to note that electrocardiogram data and follow-up laboratory data were not available in this databank. Another limitation of our study was missing data that we handled by replacing with median in continuous variables and with mode in categorical ones, which might not have been the optimal way for doing so; however, the prediction of missing data was not possible due to not having a large enough dataset. Moreover, since we tuned the threshold for classifying the groups to optimize sensitivity (recall), we were not able to assess the calibration of our models, and the probabilities in models were only used to identify the optimal threshold. Also, the fact that our data were imbalanced and we tuned our models for better prediction of AKI based on AUC using five-fold cross-validation, led to relatively lower specificities, compared to AUCs and sensitivities. This is a limitation of our study; however, it should be considered that in these types of adverse events, higher sensitivity is much favored over higher specificity since the clinician’s aim is to not miss any potentially high-risk case in terms of AKI. Also, in our study, the threshold was adjusted for higher sensitivity while in other clinical settings, it could be tuned for higher specificity based on clinical settings. Finally, despite using fivefold cross-validation in our training cohort and evaluating the models on an unseen test cohort, the lack of external validation in our study might threaten the generalizability of our findings and models.

## Conclusion

In conclusion, the ML models such as RF, LR, CB, MLP, and NB algorithms, showed an acceptable predictive performance for the risk of AKI following PCI, with RF and CB providing the greatest discriminations. Also, the most important features for the AKI prediction were detected, and LVEF demonstrated the largest coefficient in all predicting models. Therefore, it could be suggested that ML models, particularly the RF model, improve the accuracy of AKI prediction in patients undergoing PCI, which has significant implications for clinical decision-making and management to prevent AKI incidence. However, further studies are necessitated to validate the findings of the present study.

## Data Availability

The data used in this study will be made available upon reasonable request from the corresponding author.

## Abbreviations

AKI: Acute kidney injury
PCI: Percutaneous coronary intervention
ML: Machine learning
ACS: Acute coronary syndrome
NB: Naïve Bayes
LR: Logistic Regression
RF: Random Forest
CB: CatBoost
MLP: Multi-layer perception
AUC: Area under the receiver operating characteristics curve
CI-AKI: Contrast induced-AKI
STEMI: ST-elevation myocardial infarction
UA: Unstable angina
LVEF: Left ventricular ejection fraction
AF: Atrial fibrillation
FPG: Fasting plasma glucose
TG: Triglycerides
TC: Total cholesterol
LDL-C: Low-density lipoprotein cholesterol
HDL-C: High-density lipoprotein cholesterol
BMI: Body mass index
eGFR: Estimated glomerular filtration rate
Cr: Creatinine
CABG: Coronary artery bypass grafting
NSTEMI: Non-ST elevation myocardial infarction
CAG: Coronary angiography
CPR: Cardiopulmonary resuscitation
AKIN: Acute kidney injury necrosis
ESRD: End-stage renal disease
PMH: Past medical history
CI: Confidence interval
AVM: Support vector machine
KNN: K-nearest
GBM: Gradient boosting model
SHAP: SHapley Additive exPlanations
